# V-MitoSNP: visualization of human mitochondrial SNPs

**DOI:** 10.1186/1471-2105-7-379

**Published:** 2006-08-15

**Authors:** Li-Yeh Chuang, Cheng-Hong Yang, Yu-Huei Cheng, De-Leung Gu, Phei-Lang Chang, Ke-Hung Tsui, Hsueh-Wei Chang

**Affiliations:** 1 Department of Chemical Engineering, I-Shou University, 840, Taiwan; 2 Department of Electronic Engineering, National Kaohsiung University of Applied Sciences, 807, Taiwan; 3 Chang Gung Bioinformatics Center, Chang Gung Memorial Hospital, Chang Gung University, Taiwan; 4 Faculty of Biomedical Science and Environmental Biology, Kaohsiung Medical University, Kaohsiung, 80708, Taiwan

## Abstract

**Background:**

Mitochondrial single nucleotide polymorphisms (mtSNPs) constitute important data when trying to shed some light on human diseases and cancers. Unfortunately, providing relevant mtSNP genotyping information in mtDNA databases in a neatly organized and transparent visual manner still remains a challenge. Amongst the many methods reported for SNP genotyping, determining the restriction fragment length polymorphisms (RFLPs) is still one of the most convenient and cost-saving methods. In this study, we prepared the visualization of the mtDNA genome in a way, which integrates the RFLP genotyping information with mitochondria related cancers and diseases in a user-friendly, intuitive and interactive manner. The inherent problem associated with mtDNA sequences in BLAST of the NCBI database was also solved.

**Description:**

V-MitoSNP provides complete mtSNP information for four different kinds of inputs: (1) color-coded visual input by selecting genes of interest on the genome graph, (2) keyword search by locus, disease and mtSNP rs# ID, (3) visualized input of nucleotide range by clicking the selected region of the mtDNA sequence, and (4) sequences mtBLAST. The V-MitoSNP output provides 500 bp (base pairs) flanking sequences for each SNP coupled with the RFLP enzyme and the corresponding natural or mismatched primer sets. The output format enables users to see the SNP genotype pattern of the RFLP by virtual electrophoresis of each mtSNP. The rate of successful design of enzymes and primers for RFLPs in all mtSNPs was 99.1%. The RFLP information was validated by actual agarose electrophoresis and showed successful results for all mtSNPs tested. The mtBLAST function in V-MitoSNP provides the gene information within the input sequence rather than providing the complete mitochondrial chromosome as in the NCBI BLAST database. All mtSNPs with rs number entries in NCBI are integrated in the corresponding SNP in V-MitoSNP.

**Conclusion:**

V-MitoSNP is a web-based software platform that provides a user-friendly and interactive interface for mtSNP information, especially with regard to RFLP genotyping. Visual input and output coupled with integrated mtSNP information from MITOMAP and NCBI make V-MitoSNP an ideal and complete visualization interface for human mtSNPs association studies.

## Background

The human mitochondrial genome is defined by a single type of circular double-stranded DNA whose complete nucleotide sequence has been established [1] and corrected [2]. It contains 16569 bps in 37 genes. Twenty-eight of these genes are encoded by the heavy strand, and nine by the light strand. Of the 37 genes, a total of 24 specify a mature RNA product: 22 mitochondrial tRNA molecules and two mitochondrial rRNA molecules (a 23S rRNA and a 16S rRNA). The remaining 13 genes encode polypeptides, which are synthesized on mitochondrial ribosomes.

Mitochondrial DNA (mtDNA) is known for high mutation rates caused by a lack of histones, inefficient DNA repair capability, and continuous exposure to oxidative stress. It has been suggested that the mitochondrial variations are linked to the origin of humans, and play a substantial role in forensics, degenerative diseases, cancers and the aging process [3]. Mitochondrial DNA mutations are analyzed by many laboratories in order to investigate their potential role as active markers for tumorigenesis in various cancer types [4,5], e.g. cervical [6], gastric [7], ovarian [8], breast [9], colorectal [10], liver and lung cancers [11]. Many association studies for human mitochondrial genes are reported, e.g. for MT-ATP6, MT-ATP8, and MT-ND4 [12]. The study of SNPs in mtDNA has also been applied in forensic identification [13]. The substitution rate in mitochondria is typically five to 10 times higher than that of nuclear DNA [14], which has made mitochondria an attractive source for DNA polymorphism data in genetic population studies. These facts make the investigation of mtDNA polymorphisms a vital part of cancer and disease studies.

To date, several mtDNA databases have been established, i.e. mtDB [15], MITOMAP [16], GiiB-JST mtSNP [17] and MitoRes [18]. The mtDB database established in 2000 is a comprehensive database of the complete human mitochondrial genome. Included are the actual sequences, many of which have not been previously deposited in a publicly available database, such a GeneBank. MITOMAP [16] is another comprehensive database of human mtDNA variations and contains information pertaining to human evolution, diseases and cancers. GiiB-JST mtSNP [17] provides information related to the functional differences among mtSNPs. It can be used for identification of mtSNPs associated with age-related conditions, such as longevity, Parkinson's disease, and Alzheimer's disease. The mtSNPs identified in this database are also associated with conditions related to energy metabolisms, such as obesity, type 2 diabetes, and atherosclerosis. MitoRes [18] contains data on nuclear-encoded mitochondria-related products for any metazoan species, which is useful when studying mitochondrial biogenesis, and metabolic and pathological dysfunctions. However, these web-based databases don't provide enough information for complete SNP genotyping, and typically lack a convenient visualization platform. The environment of these web-based databases is not very interactive. An improvement of the visualization and interactivity could be very important for association studies related to diseases and cancers in mtSNPs. Furthermore, none of the above mentioned databases provide a correlation between the available SNP genotyping information

In the present study, we describe a new web-based visualization interface for mtSNPs, called V-MitoSNP. It provides visualization of human mtSNPs in a format convenient for association studies. The genotype information presented in V-MitoSNP is the restriction fragment of length polymorphism (RFLP), which is the most cost-effective method used in standard laboratories. V-MitoSNP identifies the restriction enzymes and their paired natural/mismatched primer sets for RFLPs in all mtSNPs and immediately presents the results in a ready-to-use format. V-MitoSNP also provides an mtSNP search capability related to the gene locus, disease, mtSNP rs# ID, genome range, and the actual sequences.

## Construction and content

### Implementation

V-MitoSNP is designed and implemented under the SQL server database system. Java Server Pages (JSP) and Java applets are used to input data and process files between the user and the application, as well as parse the data. The database structure for mtSNPs is downloaded from MITOMAP [16] with permission, and the mtSNP rs# ID is downloaded from NCBI dbSNP version b123 [19]. The mitochondrial genome sequence rCRS is also downloaded from MITOMAP [16]. The restriction enzyme database for RFLP genotyping is downloaded from REBASE version 601 [20]. The restriction enzymes are transformed into the MySOL format and saved in a local database.

### Program workflow

**Figure 1:**
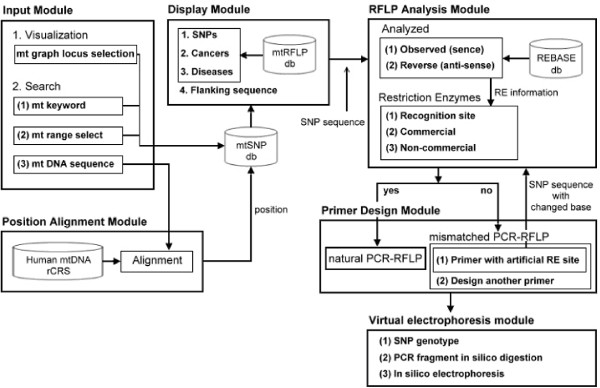
V-MitoSNP flow chart. See main text for details.

The schematic program workflow of V-MitoSNP (Figure 1) consists of six modules: (1) the input module, (2) the display module, (3) the position alignment module, (4) the RFLP analysis module, (5) the primer design module, and (6) the virtual electrophoresis module. Users can obtain the mitochondrial information via two different approaches: graphic visualization and data search. Graphic or data search using the input module is the first step when running the software. The mtDNA sequence search is programmed to match the data from the mtDNA sequence rCRS [2] in the position alignment module. After alignment, the position range for the input sequence is deposited into the local mtSNP database for future retrieval. This path is partly overlapping the mt range input. When using either the keyword search or the graphic visualization interface, results are deposited into the mtSNP database, which is constructed using data from MITOMAP [16] and chromosome MT data obtained from NCBI dbSNP [19]. Subsequently, the display module shows the RFLP availability (yes or no) of mitochondrial data after retrieval from the local mtRFLP database. The resulting SNP sequences are programmed in the RFLP analysis module. After retrieval from the local REBASE database [20], the available RFLPs for the SNP-containing sequences in both sense and antisense strands are analyzed, and finally the restriction enzyme information is displayed. The primer design for mtSNPs with an RFLP enzyme (natural primers) is different from that of mtSNPs without an RFLP enzyme (mismatched primer). Natural primers are designed for SNPs with available RFLPs. In the case of an SNP without RFLP, the mismatched primer is designed by changing the nucleotide beside the SNP in order to determine its RFLP availability. Once availability can be confirmed the opposite primer is designed. Finally, information from both the RFLP and the primer design modules is integrated in the virtual electrophoresis module. All the modules are explained below in further detail.

#### (1) Input module

**Figure 2:**
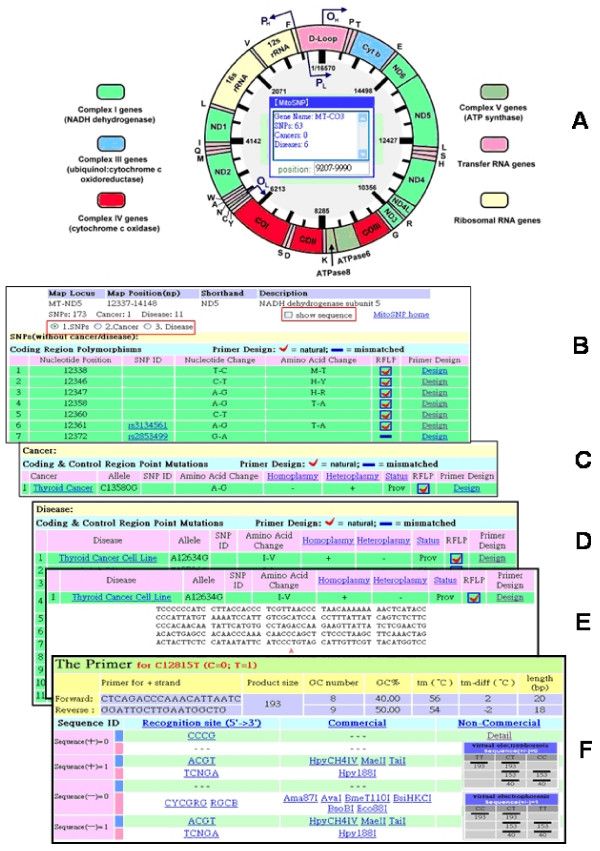
Graphic input and output format for V-MitoSNP. The mtSNP with corresponding SNP rs# ID in chromosome MT of NCBI dbSNP [19] is shown parallel if available. When the sequences are suitable for restriction enzymes, the RFLP result shows "V". In contrast, if no RFLP is available in the restriction enzyme, "X" is shown. (A) Gene selection for graphic input on map of the human mtDNA. (B) Graphic output for the ND5 gene. The mtSNPs without cancer and disease reports are shown by default. In the red box the choices for SNP information are shown. (C) Graphic output for disease-mtSNPs. (D) Graphic output for cancer-related mtSNPs. (E) An SNP with its corresponding flanking sequence (500 bp) is provided by clicking "show sequence" in the red box in Figure 2B. (F) Standard results of V-MitoSNP demonstrate detailed information, including RFLP available restriction enzymes, target site, ready-for-use primers (natural or mismatched), and virtual electrophoresis results. The C12815T mtSNP is selected as an example. The complementary SNP sequence is separated into two sequences marked "0" and "1", i.e. C = 0, T = 1 in C12815T. The sense and antisense strands are represented as"+" and "-", respectively. Both commercial and non-commercial restriction enzymes are divided into two parts marked with blue and red colors, which represent the recognition sites for endonucleases with and without degenerated nucleotides, respectively. Two virtual electrophoresis results are provided for sequence (+/-) = 0 and sequence (+/-) = 1.

**Figure 3:**
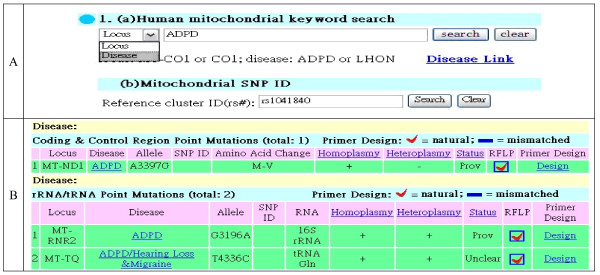
Keyword search in output module of V-MitoSNP. mtSNPs with corresponding SNP rs# ID in chromosome MT of NCBI dbSNP [19] are shown parallel if available. (A) Keyword input for locus, disease and mtSNP rs# ID. (B) Result for data input "ADPD" (disease). Homoplasmy, heteroplasmy, as well as additional SNP information (described in Figure 2) are provided.

**Figure 4:**
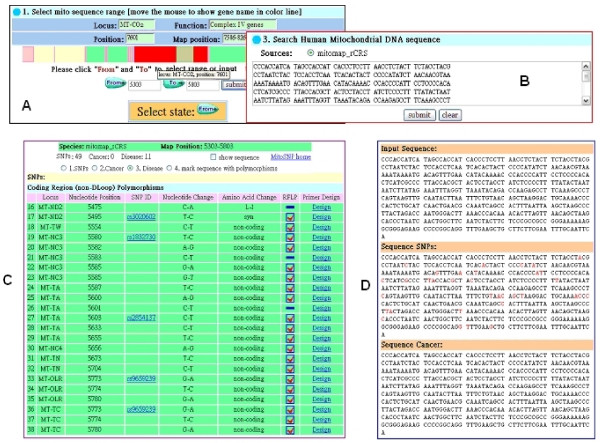
Range and sequence for the input and output in V-MitoSNP. The mtSNP with corresponding SNP rs# ID in chromosome MT of NCBI dbSNP [19] is shown parallel if available. (A) Range input by clicking (e.g., position 5303~5803 at MITOMAP sequence of rCRS). Real-time display for positional information is provided. (B) mtBLAST provides gene coverage by mtDNA sequence. (C) and (D) are common results for both (A) and (B). (C) Gene coverage for range and sequence input. Information for all SNPs within the input data range is shown in order of the nucleotide position. (D) SNPs in the sequence can be selectively shown in red color.

V-MitoSNP uses two different input formats, namely a graphic input format and a search input format. The graphic input format illustrates color-coded gene functions (Figure 2A). The green color represents genes of the complex I gene type (NADH dehydrogenase), including MT-ND1, MT-ND2, MT-ND3, MT-ND4L, MT-ND4, MT-ND5, and MT-ND6. When touching any regions on the mtDNA genome graph, a central real-time display window provides the gene name, the position range for the selected gene, the total number of SNPs within, and the number of SNPs related to cancers or diseases. In the search input format keywords, an mt range and an mtDNA sequence are acceptable. Allowed keywords can be the gene locus, a disease, and the NCBI rs# ID (Figure 3A). An input range can be selected by clicking the color-band on the graph twice using the "to" and "from" buttons, or by directly line feeding the range for the position (Figure 4A). The input of an mtDNA sequence in IUPAC format within a 10% mismatch range to the rCRS sequence is allowed by default, and can be blasted with mtBLAST, which is a gene-targeting search for an mtDNA sequence unlike NCBI BLAST (Figure 4B). (Please see the discussion for details.)

#### (2) Display module

The results of the input module are processed in the display module, which provides SNP, cancer, and disease information for the mtDNA. The displayed SNP flanking sequence is used as a template in the RFLP analysis and in the primer design modules. The RFLP availability for all mtSNPs from MITOMAP [16] and the chromosome MT of the NCBI dbSNP [19] is calculated, analyzed and stored in the mtRFLP database.

#### (3) Position alignment module

The input sequence is matched to the human mtDNA rCRS sequence [2]. Biological information is automatically provided for the matched position range.

#### (4) RFLP analysis module

The entered SNP sequences are transformed into their complementary (reverse) strands. The RFLP result for these sequences is then analyzed using the local REBASE database [20]. V-MitoSNP provides a complete list of available restriction enzymes for each mtSNP, including commercial and non-commercial restriction enzymes. The restriction enzymes are divided into two groups. Blue and red colors (Figure 2F) are used to identify the recognition sites for the restriction enzymes with and without degenerated nucleotides, respectively. The enzyme lists are updated periodically from REBASE [20].

Although most endonucleases are palindromic, the flanking sequences of the recognition site containing SNPs in both sense and antisense strands are usually different. Some recognition sites are found exclusively only in sense or antisense strands. Sometimes, the sense and antisense strands provide the same restriction enzyme for RFLP genotyping because the same recognition site is chosen in both strands. V-MitoSNP provides the RFLP availability in both sense and antisense strands labeled "+" and "-" in Figure 2F. The complementary SNP sequence is separated into two sequences marked "0" and "1" for both sense and antisense strands. If different enzymes are selected the recognition sites are cut differently. This sequence separation is designed to provide information of RFLP enzymes and their corresponding genotype.

#### (5) Primer design module

Primer design aims to construct optimal candidates. Although various primer design approaches have been proposed, the process is usually time consuming when carried out manually. V-MitoSNP is designed to provide complete primer sets for all SNPs in mtDNA, including the primer sets for natural and mismatched PCR-RFLP. Optimal primer design follows criteria described in [21,22], which include melting temperatures (Tm), length, base composition, 3'-end, repeated and self-complementary sequences and complementarity between members of a primer pair. Several primer design conditions are included in V-MitoSNP: (1) the length for the PCR product: 200~ 250 bp; (2) G or C preference at the 3'-end of the primer sets; (3) the primer length: 18 ~ 26 bp; (4) GC proportion: 40%~60%; (5) Tm: 50°C ~ 60°C; (6) Tm difference between primer sets: less than 5°C; (7) length difference between primer sets: 5 bp. Actual differences of these criteria are aggregated by weighting sums.

The primer design strategy depends on the RFLP availability for the target SNP. For SNPs with RFLP enzymes, the default primer design conditions can be used for designing the natural primer, while for SNPs without a natural RFLP enzyme a mismatched primer design is provided by V-MitoSNP. Only one nucleotide adjacent to the SNP candidate is changed randomly by the program in order to obtain the RFLP enzymes. The changed nucleotide faces preferentially away from the targeted SNP. Putting the system generated mismatch on the last two nucleotides of the primer is discouraged by the system. V-MitoSNP also tries to avoid the introduction of multiple mismatches because multiple mismatches and 3'-end mismatches in the PCR primer can potentially create problems for PCR optimization. Once found, the design of the mutagenic primer is accomplished and its opposite primer with compatible Tm and base composition will be designed with a PCR length of around 200 bp by default. This design ensures that the digested allelic fragments can be easily resolved on regular agarose gel electrophoresis.

#### (6) Virtual electrophoresis module

The natural and mismatched primer sets designed in the primer design module are blasted to the mtDNA rCRS sequence [2] to estimate the full length of the PCR. The RFLP analysis module provides RFLP enzyme information for *in silico *digestion and its corresponding SNP genotype. As described under the RFLP analysis module, the complementary SNP sequence is separated into two distinct sequences marked "0" and "1". The virtual gel patterns are different for sequences with "0" and/or "1" for sense or antisense (+ or -) strands. For example, the non-commercial restriction enzyme NcuII is cut at the C site in mtSNP at C12815T, whereas the T site in C12815T is uncut in the sequence of (+) = 0 (Figure 2F). The virtual gel pattern shows that the enzyme NcuII can digest the CC type, whereas TT cannot be digested. In contrast, enzymes listed in sequences with "1" mean that the T of C12815T is cut by HpyCH4IV and the C of C12815T is uncut. The virtual gel pattern shows that the TT type can be digested with the enzyme HpyCH4IV, but CC cannot, explaining the two distinctly different virtual gel patters produced by V-MitoSNP. After having obtained the two virtual gel patterns the *in silico *PCR-RFLP is prepared and analyzed by *in silico *electrophoresis, which shows its genotype and the corresponding PCR-RFLP length.

### Validation of primer designs

DNA extraction of human blood and a standard PCR reaction were performed as previously described [23]. To validate the functions of the designed primers in V-MitoSNP, SNPs at position 8993, 5973, 7080, 12372, 15508, and 8829 of rCRS [2] and their corresponding natural and/or mismatched primers were tested. The natural primers were the following: 8993 forward 5'-CATGGCCATCCCCTTATG-3', 8993 reverse 5'-ATGAGTACCTGGCCTGCAG-3', 5973 forward 5'-CACCTCGG AGCTGGTAAA-3', 5973 reverse 5'-TAAGGAGGCTTAGCGCTG-3', 7080 forward 5'-GAGCCCTAGGATTCATCT-3', and 7080 reverse 5'-TCTAGGGTGTAGCCAGAG-3'. The mismatched primers were: 12372 forward 5'-ACTACTATAACCACCCTAACCCTG-3', 12372 reverse 5'-TTAGGGAGAGCTGGGTTGTTTGG-3', 15508 forward 5'-GACCTCCTAGGCGACCCAGAC-3', 15508 reverse 5'-TTAGTGGGCGAAATATTATGCTTTG-3', 8829 forward 5'-ACCAACCACCCAACTATCTATAAAC-3', and 8829 reverse 5'-TGGCCTGCAGTAATGTTAGCGGT-3'. The PCR length for the SNP at 8993, 5973, 7080, 12372, 15508, and 8829 using these primers were 206, 200, 201, 221, 223, and 221 bp, respectively. Detailed PCR information was obtained online by range input. The PCR was performed under the following conditions: 94°C (1 min); 4 cycles of 94°C (15s), 64°C (15 s), 70°C (15 s); 4 cycles of 94°C (15 s), 61°C (15 s), 70°C (15 s); 4 cycles of 94°C (15 s), 58°C (15 s), 70°C (15 s); 60 cycles of 94°C for (15 s), 55°C (15 s), 70°C (15 s); 94°C (1 min) and 60°C (5 min). The PCR results were confirmed by 1.5% agarose electrophoresis (stained with ethidium bromide).

## Utility

### Graphic input and output visualization

Users can select a gene of interest on the mitochondrial genome graph, which is subdivided into different regions for each gene, simply by clicking on it (Figure 2A). Genes with similar functions are shown in the same colors. By selecting a certain region of the graph real-time information about the gene name, the total number of genes, and cancer- and disease-related SNPs within the selected genes can be provided. The visualized output data of V-MitoSNP is plotted in Figures 2B~2F in an overlapping manner. The MT-ND5 gene was chosen as an example to show the general results for the gene input. In Figure 2B, SNP information for the input gene is shown, including its map locus, map position, shorthand, description, SNP number with or without cancer and disease information, sequence of the selected SNP, NCBI rs# ID, nucleotide position, nucleotide change, amino acid change, RFLP availability and the ready-for-use primers with their respective virtual electrophoresis information. The total number of mtSNPs, cancer-related mtSNPs, and disease-related mtSNPs are shown in the red box in Figure 2B. The red box contains three available choices for SNP information: mtSNPs not related to cancers/diseases, cancer-related mtSNPs, and mtSNPs related to other diseases. Information pertaining to mtSNPs without the report for cancer and disease in MITOMAP [16] is shown by default (Figure 2B). Disease- and cancer-related mtSNPs are shown in Figure 2C and Figure 2D, respectively. Both Figure 2C and Figure 2D provide extra information in addition to Figure 2B, e.g. homoplasmy and heteroplasmy. V-MitoSNP also shows the full name of cancers and diseases via a hyperlink to MITOMAP. When clicking the check box "show sequence" in Figure 2B, each SNP with its corresponding flanking sequence (500 bp) is provided in Figure 2E for primer design if needed. Natural primers are designed by V-MitoSNP, and their virtual electrophoresis results are shown in Figure 2F.

In Figure 2F, sequence (+/-) is used to represent the sense and antisense sequences. Sequence (+/-) = 0 and sequence (+/-) = 1 are used to represent the sequence with C in C12815T and T in C12815T, respectively. In sequence (+) = 0, the enzyme with the recognition site CCCG is provided in detail for a noncommercial enzyme, i.e. NcuII and Sth132I. In sequence (-) = 0, enzymes with recognition sites CYCGRG and RGCB are provided for commercial enzymes like Ama87I, AvaI, BmeT110I, BsiHKCI, BsoBI, Eco88I. Noncommercial enzymes can be shown in detail via a hyperlink. The results shown in the figures suggest that the same SNP (= 0) shown in sense and antisense strands can correspond to different RFLP enzymes. In the sequence (+/-) = 1, enzymes with recognition sites ACGT and TCNGA are provided for commercial enzymes like HpyCH4IV, MaeII, TaiI, Hpy188I, and noncommercial enzymes can again be shown in detail by clicking on a hyperlink. The results for the sequence (+/-) = 1 suggest that under some circumstances the RFLP enzymes in both sense and antisense strands can be identical. Similar functions can be displayed for mismatched primers, except that only the sense strand is presented (not shown in the figures, please check results on the V-MitoSNP website).

The virtual RFLP pattern after the *in silico *enzyme digestion is dependent on the sequence trait, meaning the sense or antisense strands. In Figure 2F, V-MitoSNP provides two kinds of virtual gel patterns for the sequence (+/-) = 0 and the sequence (+/-) = 1. The *in silico *PCR-RFLP can be prepared and analyzed by *in silico *electrophoresis, which shows the genotype and the corresponding PCR-RFLP length pattern. The obtained virtual gel patterns and electrophoresis results facilitate RFLP genotyping enormously, since they contain information about the digested fragment length and the corresponding SNP genotypes. Information for both commercial and non-commercial restriction enzymes is provided for recognition sites with and without degenerated nucleotides. The results shown in Figure 2B~2F are also typical for output formats of a data search and of a range and sequence search, both of which are not shown in the Figures 3 and 4 for brevity.

### Data search input and output

V-MitoSNP provides a keyword search function for convenience. Threekinds of keyword inputs, namely locus (gene name), disease and mtSNP rs# ID can be selected (Figure 3A). In Figure 3B the disease ADPD is shown as an output result. In addition to the common RFLP information described in Figure 2, homoplasmy, heteroplasmy and status are also provided. The disease ADPD is related to several genes, including MT-ND1 (coding & control region in point mutations), MT-RNR2 and MT-TQ (rRNA/tRNA in point mutation). The results of the search function of V-MitoSNP indicate that relationship, and provide a hyperlink for further information.

The results of the mtSNP rs# ID output (not shown for brevity) can also constitute a convenient manner for systematic RFLP genotyping association studies. The connection between the SNP in the rCRS sequence [2] and the chromosome MT in NCBI dbSNP [19] is also indicated. The SNP in the rCRS sequence obtained from the NCBI rs # ID record is shown parallel if available.

### Range & sequence input and output

The range position is selectable by clicking the mtDNA color-band graph, and a real-time display for positional information is provided. In Figure 4A, positions 5303~5803 on the MITOMAP sequence of rCRS are chosen. The sequence in this range is used by V-MitoSNP for input in mtBLAST (Figure 4B). Figures 4A and 4B cover genes within the input range and the respective output results are shown in Figures 4C and 4D. Information for all mtSNPs within the input data range is shown in the order of their nucleotide positions (Figure 4C). All mtSNPs contained within the sequence can be displayed with or without cancer/disease information, and are highlighted in red color.

### Validation of primer designs

**Figure 5:**
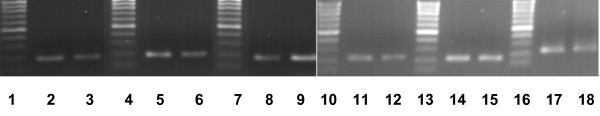
The mtSNP primer validation for V-MitoSNP. To validate the V-MitoSNP designed primers, the corresponding natural and/or mismatched primers for mtSNPs at position 8993, 5973, 7080, 12372, 15508, and 8829 of rCRS [2] are tested by PCR. Rows 1, 4, 7, 10, 13, and 16 are 100 bp DNA markers. The brightest band is 500 bp and the band at the bottom is 100 bp. Primer sets for 8993, 5973 and 7080 are natural primers, which are shown in duplicate at rows 2/3, rows 5/6, and rows 8/9, while 12372, 15508, and 8829 are mismatched primers, which are shown in duplicate at rows 11/12, rows 14/15 and rows 17/18, respectively.

Natural primers (positions at 8993, 5973, and 7080 of rCRS) and mismatched primers (positions at 12372, 15508, and 8829 of rCRS) are designed for a successful PCR (Figure 5). The sequences of the PCR products are confirmed *in silico *and contain RFLP enzymes therein (for RFLP availability, please go to the V-MitoSNP website).

## Discussion

V-MitoSNP was compared to some existing mitochondrial analysis tools, i.e. mtDB [15], MITOMAP [16], GiiB-JST mtSNP [17] and MitoRes [18]. The results indicate that V-MitoSNP is highly efficient and more informative than these other tools, especially when taking its comprehensive input and output data, such as RFLP enzyme availability, flanking sequence for selected SNP, mtBLAST, natural and mismatched primer design, and virtual electrophoresis into account. The tools that were compared to V-MitoSNP only support simple browser functions so that a user cannot obtain the complete set of data needed for comprehensive SNP genotyping. In contrast, V-MitoSNP processes graphic and data input for mtSNP analysis and retrieval. The results are presented in a user-friendly and highly structured way, thus simplifying the RFLP genotyping process considerably.

The complete RFLP enzyme list in V-MitoSNP provides for reliable and robust genotyping assays. The six primers (position of 8993, 5973, 7080, 12372, 15508, and 8829 of rCRS) provided and tested by V-MitoSNP were proven to be successful by actual agarose gel electrophoresis (Figure 5). Actually, many SNP RFLP tools do already exist, such as NEBcutter [24], PIRA-PCR Designer [25], SNP cutter [26], and SNPselector [27]. Unfortunately, the designed primer assays are usually not very effective because only SNP rs # and sequence inputs are acceptable. In the most commonly used mtDNA databases mtDB [15], MITOMAP [16], and GiiB-JST mtSNP [17] however, the polymorphism data of the mtDNA is not represented as SNP rs # (or as the SNP ID in NCBI dbSNP) [19]. V-MitoSNP merges data for the polymorphisms and the SNP rs# IDs with chromosome MT data in NCBI dbSNP [19]. The mtSNP rs# ID is also acceptable as a search input in V-MitoSNP. Commercial and non-commercial restriction enzymes for recognition sites with and without degenerated nucleotides are included, and the RFLP availability for mtSNPs is provided, extending the scope of information gathered by V-MitoSNP.

V-MitoSNP provides an almost complete RFLP restriction enzyme list, which includes corresponding primer sets for all mtSNPs from MITOMAP. MITOMAP lists 1969 mtSNPs. V-MitoSNP provides 1636 natural and 333 mismatched RFLP designed enzymes and primer sets. The mtSNPs for which RFLP enzymes and primers cannot be provided number only 18. In NCBI dbSNP (version b123) 118 mtSNPs are reported. For these, V-MitoSNP provides 105 natural and 13 mismatched RFLP enzymes and primer sets. For every mtSNP in chromosome MT listed in NCBI dbSNP complete information for RFLP genotyping is provided. Periodic updates for V-MitoSNP are planned, which will include the latest versions of NCBI dbSNP and the up-to-date information contained therein. The rate for successful enzyme and primer design for RFLP in all mtSNPs was at least 99.1% when using V-MitoSNP.

We found it convenient to manage RFLP enzymes and the corresponding primers in the graphically displayed and ready-for-use format provided by V-MitoSNP. The graphic display of the results and the organized data structure are features that set V-MitoSNP apart from the other tools tested here. V-MitoSNP presents size data of amplicons, digested allelic fragments and signature fragments in virtual electrophoresis (Figure 2F).

This data is important as a guide for evaluating genotyping results. Although PCR-RFLP is not generally recognized as a high-throughput SNP genotyping method, it does have its advantages and still plays an important role in many small laboratories due to its cost effectiveness. V-MitoSNP was specifically developed as a tool to assist investigators who are using PCR-RFLP when performing SNP genotyping in mtDNA.

V-MitoSNP provides a gene-specific homologue search of mtDNA sequences for mtBLAST. Even if the sequence used covers several genes in the mtDNA, V-MitoSNP will still shows all genes within the sequence, as well as the SNP genotyping information. The input sequence from nucleotide 5303 to 5803 in rCRS [2] (Figure 2D) outputs the genes MT-ND2, MT-TW, MT-NC3, and MT-TA (in Figure 3C). In contrast, the results of NCBI BLASTn [28] using the nr database show different isolates for the complete *Homo sapiens *mitochondrion genome (100 hits). A gene name output cannot be provided. The same results were obtained by analysis with the Biology Workbench 3.2 at the San Diego Supercomputer Center [29] using the *Homo sapiens *mitochondrion genome (hs_ref_chrMT.na) database. To our knowledge, V-MitoSNP is the first software that provides a gene-targeting function to BLAST mtDNA, although a score and E value are not included.

## Conclusion

V-MitoSNP presents ready-for-use mtSNP information related to diseases and cancers in a graphic, user-friendly and structured manner. It is convenient for use in mtSNP association studies and simplifies these considerably. Primer set and its corresponding RFLP restriction enzyme are provided. Visual input and output coupled with integrated mtSNP information from MITOMAP and NCBI make V-MitoSNP an ideal and complete visualization interface for human mtSNPs association studies.

## Availability and requirements

Project name: V-MitoSNP: visualization of human mitochondrial

SNPs Project home page: 

Operating system(s): Microsoft Windows XP

Programming language: Java

Other requirements: JSP 2.0, Servlet 2.4, Tomcat 5.5, SQL server 2000, MySQL 4.0

License: none for academic users.

For any restrictions regarding the use by non-academicians please contact the corresponding author.

## Abbreviations

mt, Mito, Mitochondria

SNP, Single Nucleotide Polymorphism

rCRS, revised Cambridge Reference Sequence

NCBI, National Center for Biotechnology Information

RFLP, Restriction Fragment Length Polymorphism

REBASE, Restriction Enzyme Database

PCR, Polymerase Chain Reaction

chromosome MT, Mitochondrial chromosome

## Authors' contributions

L-YC provided the biochemistry background, introduced the bioinformatics for mitochondrial SNPs and wrote the manuscript. Both L-YC and C-HY instructed Y-HC in writing the software algorithm. P-LC and K-HT participated in the earlier development of the program. D-LG tested the program and validated the primers designed by V-MitoSNP. H-WC coordinated and oversaw this study.
